# The ‘known’ genetic potential for microbial communities to degrade organic phosphorus is reduced in low‐pH soils

**DOI:** 10.1002/mbo3.474

**Published:** 2017-04-16

**Authors:** Ian D. E. A. Lidbury, Tandra Fraser, Andrew R. J. Murphy, David J. Scanlan, Gary D. Bending, Alexandra M. E. Jones, Jonathan D. Moore, Andrew Goodall, Mark Tibbett, John P. Hammond, Elizabeth M. H. Wellington

**Affiliations:** ^1^ School of Life Sciences University of Warwick Coventry West Midlands United Kingdom; ^2^ School of Agriculture, Policy, and Development University of Reading Whiteknights United Kingdom; ^3^ The Genome Analysis Centre Norwich Research Park Norwich United Kingdom; ^4^ Southern Cross Plant Science Southern Cross University Lismore Australia

**Keywords:** Acid phosphatase, alkaline phosphatase, metagenomics, microbial community, microbial diversity, soil

## Abstract

In soil, bioavailable inorganic orthophosphate is found at low concentrations and thus limits biological growth. To overcome this phosphorus scarcity, plants and bacteria secrete numerous enzymes, namely acid and alkaline phosphatases, which cleave orthophosphate from various organic phosphorus substrates. Using profile hidden Markov modeling approaches, we investigated the abundance of various non specific phosphatases, both acid and alkaline, in metagenomes retrieved from soils with contrasting pH regimes. This analysis uncovered a marked reduction in the abundance and diversity of various alkaline phosphatases in low‐pH soils that was not counterbalanced by an increase in acid phosphatases. Furthermore, it was also discovered that only half of the bacterial strains from different phyla deposited in the Integrated Microbial Genomes database harbor alkaline phosphatases. Taken together, our data suggests that these ‘phosphatase lacking’ isolates likely increase in low‐pH soils and future research should ascertain how these bacteria overcome phosphorus scarcity.

## 1 Introduction

In soil, total phosphorus (P) concentrations typically vary between 100 and 3,000 mg kg^−1^ (Hedley et al. 1995; Mengel 1997), of which 30%–60% is in the form of complex organic P esters (White & Hammond, 2008). However, plants can only acquire simple orthophosphate (Pi), which is frequently found at low concentrations in soils (<10 μmol L^‐1^) (White & Hammond, 2008). Therefore, P is often a limiting nutrient for global crop production and subsequently inorganic rock phosphate is applied in large quantities with deleterious economic and environmental consequences (Vance, Uhde‐Stone, & Allan, 2003; Cordell, Drangert, & White, 2009; White & Hammond, 2008, 2009).

Microorganisms can have a beneficial effect on crop production, partly through the liberation of unavailable P in the rhizosphere and surrounding soil (Rodríguez & Fraga, 1999). Various studies have revealed that upon depletion of extracellular Pi bacteria can undergo a regulatory and physiological response resulting in the secretion of various exoproteins involved in liberating and binding Pi (Hirani, Suzuki, Murata, Hayashi, & Eaton‐Rye, 2001; Rittmann, Sorger‐Herrmann, & Wendisch, 2005; Monds, Newell, Schwartzman, & O'Toole, 2006; Antelmann, Scharf, & Hecker, 2000; Lidbury et al., 2016). Through this process, numerous reports have revealed that various phosphatases play an important role in the bioavailability of P in soils (Nannipieri, Newell, Giagnoni, Landi, & Renella, 2011). Usually, the most heavily secreted exoenzymes are alkaline phosphatases (APases), which cleave Pi from a plethora of complex organic P monoesters and diesters. The latter compounds account for the bulk (up to 90%) of organic P in soils (Condron, Turner, & Cade‐Menun, 2005; Lidbury et al., 2016; Santos‐Beneit, 2015; Zaheer, Morton, Proudfoot, Yakunin, & Finan, 2009). APases encompass a wide genetic diversity including PhoA (Roy, Ghosh, & Das, 1982), PhoX (Sebastian & Ammerman, 2009) and PhoD (Eder, Shi, Jensen, Yamane, & Hulett, 1996) types. APases are secreted through various mechanisms, including the twin‐arginine translocation (TAT) pathway (Putker et al., 2013) or sec pathway (PhoA) (Angelini et al., 2001). Mutagenesis of *phoX* almost entirely abolishes *para*‐nitrophenyl phosphate degradation capacity (Monds et al., 2006), the substrate most frequently used to determine in situ soil phosphatase activity. Similarly, both PhoD and PhoA have been shown to be highly active against *para*‐nitrophenyl phosphate (Rodriguez et al., 2014; Yang & Metcalf, 2004). APases are thought to be partly responsible for the ‘P‐fertilisation’ effect seen when inoculating soils with plant growth‐promoting bacteria (PGPB) (Condron et al., 2005; Rodríguez & Fraga, 1999).

## 2 Experimental procedures

### 2.1 Bioinformatic analyses

For each protein analyzed, between 50 and 80 sequences from a range of phylogenetically distinct soil bacteria were downloaded from the Integrated Microbial Genomes database (IMG/JGI) (https://img.jgi.doe.gov/). To identify these homologs, a combination of BLASTP and function searches (IMG search option) using the conserved PFAM domains for each protein were performed. Downloaded sequences were aligned using MUSCLE and HMM profiles were constructed using the hmmbuild function in HMMER (Eddy, 1998).

The eight metagenomes were downloaded from the EBI metagenomics portal under the project code ERP001068 (Title: Functional diversity of soil microbes across environmental gradients). The eight sample IDs for each metagenome are as follows: ERS078132, ERS078133, ERS078134, ERS078135, ERS078136, ERS078137, ERS078138, ERS078139 corresponding to CS1, CS179, CS864, CS922, CS78, CS251, CS511, CS1053 sites of the UK countryside survey, respectively. Information regarding the taxonomic profile of each site (based on the 16S rRNA gene) can be obtained directly from the EBI metagenomics portal. For each site, both FASTA files for the ‘predicted CDS’ and ‘predicted CDS without annotation’ were downloaded.

Each site was screened for the abundance of each functional gene using the hmmsearch option in HMMER and using easel to generate output files. For determining the abundance of a given functional gene at each site, the method used by Howard, Sun, Biers, and Moran (2008) was adapted. Briefly, the raw number of hits at each site was normalized to RecA by taking the ratio of the length (amino acid) of each given functional protein against the length (amino acid) of RecA. Four single copy housekeeping genes, RpoB, AtpB, GyrB, and SucD were also used. The mean number of counts retrieved for each enzyme was used to determine the ‘average genome equivalent’ for each site. Next, the ratio of the number of hits related to phosphorus‐scavenging enzymes against each genome equivalent was calculated to provide the number of bacteria containing a given enzyme at each site.

To assess the diversity of alkaline phosphatases and GyrB, manually curated databases were established for each gene by downloading all the known homologs present in the IMG/JGI database. A BLASTP search was performed using a relatively relaxed stringency (e‐10). To confirm the validity of the hits retrieved from hmmsearches, individual sequences retrieved by hmmsearches were used as queries in BLASTP searches using the nr database located on the National Centre for Biotechnology Information server (www.ncbi.nlm.nih.gov/BLAST.cgi). Higher taxonomic ranks were retrieved using the NCBI taxonomy tool. The data were visualized using the Krona tools package (Ondov et al. 2011).

## 3 Results and Discussion

To determine the ‘genetic potential’ of microbial communities to remineralize organic forms of P, we screened eight metagenomes (https://www.ebi.ac.uk/metagenomics/) from geographically distinct locations across the UK landscape (Griffiths et al., 2011). Four sites were considered low‐pH soils (4.12–4.37), and four high‐pH soils (8.04–8.46). Profile hidden Markov modeling (HMM) searches (Eddy, 1998) were conducted for each APase (PhoX, PhoD, PhoA), the nonspecific class A, class B, class C acid phosphatases (ACPases) (Gandhi & Chandra, 2012; Thaller, Berlutti, Schippa, Lombardi, & Rossolini, 1994), a number of other P‐scavenging enzymes and several housekeeping genes (see Table S1). The number of bacteria containing P‐scavenging genes was determined according to Howard et al. (2008). Based on 16S rRNA gene diversity, there were no significant changes (*T*‐test, *p* > .06) in the dominant phyla (Actinobacteria, Proteobacteria, Firmicutes, Acidobacteria) between low‐pH and high‐pH soils (Figure S1). The diversity of GyrB at the phylum and class levels was also comparable (Figure S4) between low‐ and high‐pH soils. Furthermore, there were no major differences in the broad functional capabilities (based on GO terms) between the high‐pH and low‐pH sites (Figure S2).

**Figure 1:**
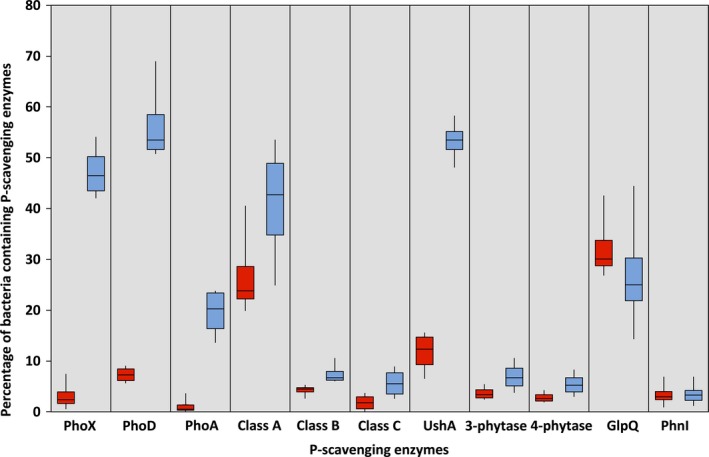
The abundance of various P‐scavenging enzymes detected in the eight metagenomes retrieved from soils with contrasting pH values (red, low pH; blue, high pH). The percentage of bacteria containing phosphatases was calculated assuming a copy number of one per cell. A reduction in the three alkaline phosphatases was observed in low‐pH soils while there was no concurrent increase in acid phosphatases Abbreviations: PhoX, PhoD, PhoA, alkakine phosphatases; classA/B/C, nonspecific acid phosphatases; UshA, 5′ nucleotidase, GlpQ, glycerolphosphodiester phosphodiesterase; PhnI, carbon‐phosphorus lyase complex subunit I

**Figure 2:**
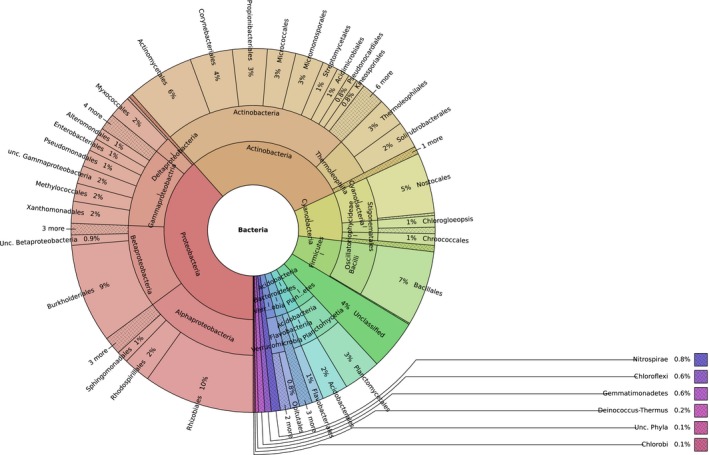
Combined taxonomic diversity of the three alkaline phosphatases (PhoX, PhoD, PhoA) in the metagenomes retrieved from all four high‐pH sites (CS922, CS78, CS251, CS511). The chart was generated using the KronaTools software package (Ondov et al., 2011)

However, there was a significant reduction (*T*‐test, unpaired, *p* < .05) in the various APases (PhoX, PhoD, PhoA) detected in low‐pH soils compared to high‐pH soils. In high‐pH soils, 47%, 56%, and 20% of bacteria possessed genes encoding PhoX, PhoD, and PhoA, respectively, whereas in low‐pH soils, only 3%, 7% and 1% of bacteria contained these genes, respectively (Figure 1A). Unexpectedly, the number of bacteria containing class A and C ACPases was similar (*T*‐test, unpaired, *p* > .05) between all sites while the number of bacteria containing class B ACPases actually decreased (*T*‐test, unpaired, *p* = .046) in low‐pH soils (Figure 1A). Besides the nucleotidase, UshA (Rittmann et al., 2005), which also showed a significant increase in high‐pH soils, the abundance of several other enzymes involved in organic P scavenging (phytases, phosphodiesterases) did not change (*T*‐test, unpaired, *p* > .05) between low‐pH and high‐pH soils. The diversity of the three APases was comparable to that of GyrB and the 16S rRNA gene (Figure 2 and S3). For example, the majority of APase sequences (63%–73%) were related to the Proteobacteria, Actinobacteria, and Firmicutes.

Based on our curated databases for APases, the majority of sequences found in the soils analyzed here had a mean amino acid identity of 69% to those found in the Integrated Microbial Genomes database at the Joint Genome Institute (IMG/JGI). However, due to a lack of biochemical and biophysical data on the majority of APases found in less‐studied bacteria that make up our curated database, for example, members of the *Betaproteobacteria* or *Bacteroidetes*, it remains to be determined whether all of the sequences related to the various APases represent *bona fide* APases. Future work should ascertain whether or not predicted APases found in genomic databases, having significant sequence divergence from the relatively few characterized APases, function in a similar manner to those studied to date.

To determine the number of sequenced bacterial genomes possessing APases, we scrutinized all the genomes (status, ‘finished’) deposited in the IMG/JGI for the presence of each APase. It was discovered that approximately half of the strains related to the Proteobacteria (1510/2244), Actinobacteria (257/494), Firmicutes (471/1052), and Cyanobacteria (66/99) possess at least one of the three APases (PhoX, PhoA, PhoD). Furthermore, only 165/258 genomes (status, finished) tagged with ‘soil’ and 37/45 genomes (status, finished) tagged with ‘rhizome/rhizoplane’ under the filter ‘ecosystem type’ harbor one of the three APases, revealing that a number of bacteria exist that do not possess the typical phosphatases associated with overcoming Pi limitation (Lidbury et al., 2016; Santos‐Beneit, 2015).

Together, this analysis revealed that low‐pH soils have a marked reduction in their ‘known genetic potential’ to remineralize organic P. In a previous study, using a targeted amplicon approach, soil pH was also shown to be a driver of *phoD* diversity (Ragot, Kertesz, & Bünemann, 2015). Whether this reduction in genetic potential found in low‐pH soils equates to a reduction in P remineralization capabilities warrants further investigation. Although APases have a pH optimum between 9 and 11, PhoX showed activity against 79 phosphomonoesters at pH 7.5 (Zaheer et al., 2009) and PhoD functions well at pH 8 (Rodriguez et al., 2014). However, it is likely that these promiscuous enzymes do not function in soils at such low‐pH values. Considering that the pH optimum of ACPases is much lower (pH 4.8–7) (Rossolini et al., 1998), it was surprising that there was no observed increase in the genes encoding these enzymes in low‐pH soils.

Low‐pH soils may select for microbial communities containing bacteria lacking ‘known’ APases (PhoX, PhoD, PhoA), but which possess as‐yet‐unidentified phosphatases, rather than a total loss of non‐specific phosphatases from bacterial genomes. This hypothesis is indirectly supported by evidence that numerous bacterial strains deposited in genome banks lack characterized APases. Given the fact that soils harbor a tremendous amount of genetic diversity (Torsvik & Øvreås, 2002) and that multiple Phyla inhabit the rhizosphere (Bulgarelli, Schlaeppi, Spaepen, van Themaat, & Schulze‐Lefert, 2013; Bulgarelli et al., 2012), our knowledge of the P‐scavenging abilities of soil bacteria remains relatively poor. Clearly our understanding of the microbial response to Pi limitation is limited to studies focusing on easily culturable strains. Future studies should focus on APase‐lacking isolates to determine their P mineralization capabilities and thus their capacity to overcome P scarcity in soils. This should include the isolation and cultivation of various bacterial strains from low‐pH soils to determine if they still elicit phosphatase activity or conversely, are reliant solely on various forms of inorganic P.

## Conflict of Interest

The authors declare no conflict of interest.
