# Supplementary material for: The ‘known’ genetic potential for microbial communities to degrade organic phosphorus is reduced in low‐pH soils
Source: Microbiologyopen. 2017 Apr 16;6(4):e00474. doi: 10.1002/mbo3.474 (PMC5552915; doi:10.1002/mbo3.474)
Supplement: Supplementary file 1 [file MBO3-6-na-s001.docx]

**Supplementary information**

Table S1. Counts retrieved from each metagenome for each enzyme.

|  |  |  | RecA |  | GyrB |  | AtpD |  | RpoB |  | SucD |  |  |
| --- | --- | --- | --- | --- | --- | --- | --- | --- | --- | --- | --- | --- | --- |
| Metagenome ID | Site ID | Sites | Raw counts | Size norm.^A^ | Raw counts | Size norm.^A^ | Raw counts | Size norm.^A^ | Raw counts | Size norm.^A^ | Raw counts | Size norm.^A^ | Average Genome Equivalents |
| ERS078132 | CS1 | LowpH1 | 66 |  | 109 | 58.05 | 140 | 105.87 | 158 | 45.66 | 48 | 55.84 | 66.28 |
| ERS078133 | CS179 | LowpH2 | 96 |  | 179 | 95.34 | 215 | 162.59 | 254 | 73.40 | 115 | 133.79 | 112.22 |
| ERS078134 | CS864 | LowpH3 | 140 |  | 214 | 113.98 | 300 | 226.87 | 354 | 102.31 | 162 | 188.48 | 154.32 |
| ERS078135 | CS922 | LowpH4 | 142 |  | 277 | 147.53 | 248 | 187.55 | 345 | 99.70 | 118 | 137.28 | 142.81 |
| ERS078136 | CS78 | HighpH1 | 110 |  | 236 | 125.70 | 189 | 142.93 | 290 | 83.81 | 102 | 118.67 | 116.22 |
| ERS078137 | CS251 | HighpH2 | 110 |  | 262 | 139.54 | 245 | 185.28 | 403 | 116.47 | 158 | 183.82 | 147.02 |
| ERS078138 | CS511 | HighpH3 | 139 |  | 315 | 167.77 | 289 | 218.55 | 460 | 132.94 | 157 | 182.66 | 168.18 |
| ERS078139 | CS1053 | HighpH4 | 160 |  | 332 | 176.82 | 309 | 233.68 | 441 | 127.45 | 158 | 183.82 | 176.35 |
|  |  |  | PhoX |  |  | PhoD |  |  | PhoA |  |  |  |  |
| Metagenome ID | Site ID | Sites | Raw counts | Size norm.^A^ | % cells | Raw counts | Size norm.^A^ | % cells | Raw counts | Size norm.^A^ | % cells |  |  |
| ERS078132 | CS1 | LowpH1 | 5 | 5.08 | 7.67 | 6 | 3.83 | 5.78 | 0 | 0 | 0 |  |  |
| ERS078133 | CS179 | LowpH2 | 1 | 0.56 | 0.50 | 15 | 9.58 | 8.53 | 1 | 0.73 | 0.65 |  |  |
| ERS078134 | CS864 | LowpH3 | 6 | 4.52 | 2.92 | 22 | 14.94 | 9.68 | 1 | 0.73 | 0.47 |  |  |
| ERS078135 | CS922 | LowpH4 | 3 | 2.82 | 1.97 | 14 | 8.94 | 6.26 | 7 | 5.15 | 3.60 |  |  |
| ERS078136 | CS78 | HighpH1 | 109 | 61.59 | 52.99 | 123 | 78.56 | 67.59 | 36 | 26.498 | 22.79 |  |  |
| ERS078137 | CS251 | HighpH2 | 129 | 72.89 | 49.57 | 121 | 77.28 | 52.56 | 35 | 25.76 | 17.52 |  |  |
| ERS078138 | CS511 | HighpH3 | 131 | 74.02 | 44.01 | 145 | 92.61 | 55.06 | 31 | 22.81 | 13.56 |  |  |
| ERS078139 | CS1053 | HighpH4 | 131 | 74.02 | 41.97 | 140 | 89.42 | 50.70 | 57 | 41.95 | 23.78 |  |  |
|  |  |  |  |  |  |  |  |  |  |  |  |  |  |
|  |  |  | Class A |  |  | Class B |  |  | Class C |  |  |  |  |
| Metagenome ID | Site ID | Sites | Raw counts | Size norm.^A^ | % cells | Raw counts | Size norm.^A^ | % cells | Raw counts | Size norm.^A^ | % cells |  |  |
| ERS078132 | CS1 | LowpH1 | 14 | 18.02 | 27.18 | 2 | 3.01 | 4.54 | 2 | 2.54 | 3.84 |  |  |
| ERS078133 | CS179 | LowpH2 | 42 | 54.06 | 48.17 | 2 | 3.01 | 2.68 | 0 | 0 | 0 |  |  |
| ERS078134 | CS864 | LowpH3 | 36 | 46.34 | 30.02 | 5 | 7.53 | 4.87 | 1 | 1.27 | 0.82 |  |  |
| ERS078135 | CS922 | LowpH4 | 25 | 32.18 | 22.53 | 5 | 7.53 | 5.27 | 3 | 3.82 | 2.67 |  |  |
| ERS078136 | CS78 | HighpH1 | 48 | 61.78 | 53.16 | 8 | 12.04 | 10.36 | 8 | 10.18 | 8.76 |  |  |
| ERS078137 | CS251 | HighpH2 | 71 | 91.39 | 62.16 | 7 | 10.54 | 7.17 | 3 | 3.82 | 2.59 |  |  |
| ERS078138 | CS511 | HighpH3 | 57 | 73.37 | 43.62 | 7 | 10.54 | 6.26 | 5 | 6.36 | 3.78 |  |  |
| ERS078139 | CS1053 | HighpH4 | 39 | 50.20 | 28.46 | 7 | 10.54 | 5.97 | 10 | 12.73 | 7.22 |  |  |
|  |  |  | GlpQ |  |  | UshA |  |  | 3-  phytase |  |  |  |  |
| Metagenome ID | Site ID | Sites | Raw counts | Size norm.^A^ | % cells | Raw counts | Size norm.^A^ | % cells | Raw counts | Size norm.^A^ | % cells |  |  |
| ERS078132 | CS1 | LowpH1 | 20 | 17.67 | 26.65 | 5 | 4.418 | 6.66 | 4 | 3.71 | 5.60 |  |  |
| ERS078133 | CS179 | LowpH2 | 69 | 60.96 | 54.32 | 19 | 16.78 | 14.95 | 3 | 2.78 | 2.48 |  |  |
| ERS078134 | CS864 | LowpH3 | 56 | 49.48 | 32.06 | 19 | 16.78 | 10.87 | 5 | 4.64 | 3.01 |  |  |
| ERS078135 | CS922 | LowpH4 | 45 | 39.76 | 27.84 | 25 | 22.09 | 15.46 | 6 | 5.57 | 3.90 |  |  |
| ERS078136 | CS78 | HighpH1 | 89 | 78.64 | 67.66 | 68 | 60.08 | 51.69 | 13 | 12.08 | 10.39 |  |  |
| ERS078137 | CS251 | HighpH2 | 110 | 97.19 | 66.10 | 81 | 71.57 | 48.68 | 6 | 5.57 | 3.79 |  |  |
| ERS078138 | CS511 | HighpH3 | 158 | 139.61 | 83 | 111 | 98.08 | 58.31 | 10 | 9.29 | 5.52 |  |  |
| ERS078139 | CS1053 | HighpH4 | 157 | 138.72 | 78.66 | 108 | 95.42 | 54.11 | 15 | 13.94 | 7.90 |  |  |

^A^ Normalised against the length of RecA (373 amino acids).

**Figure Legends**

**Figure S1**

The mean relative abundance (n=4) of different phyla, based on the 16S rRNA gene, present in low pH and high pH soils across the UK landscape. Values were obtained from the EBI metagenomics portal.

**Figure S2**

The relative abundance of the most frequently occurring GO terms, either related to molecular function (A), biological processes (B), or cellular component (C). The Figure was obtained from the EBI metagenomics portal using the ‘comparison tool’ option. Low pH soils are ERR059346-49 and the high pH soils are ERR059350-53.

**Figure S3**

The diversity of PhoX **(A)**, PhoD **(B)**, PhoA **(C)** sequences retrieved from the metagenomes of the four high pH soils. All four sites were concatenated into a single file. Plot were constructed using the Krona Tools software package (Ondov et al 2011).

**Figure S4**

The diversity of GyrB sequences retrieved from the metagenomes of the four high pH soils **(A)** and the four low pH soils **(B)**. All four sites for each pH were concatenated into a single file. Plot were constructed using the Krona Tools software package (Ondov et al 2011).

Figure S1

Figure S2

(A)


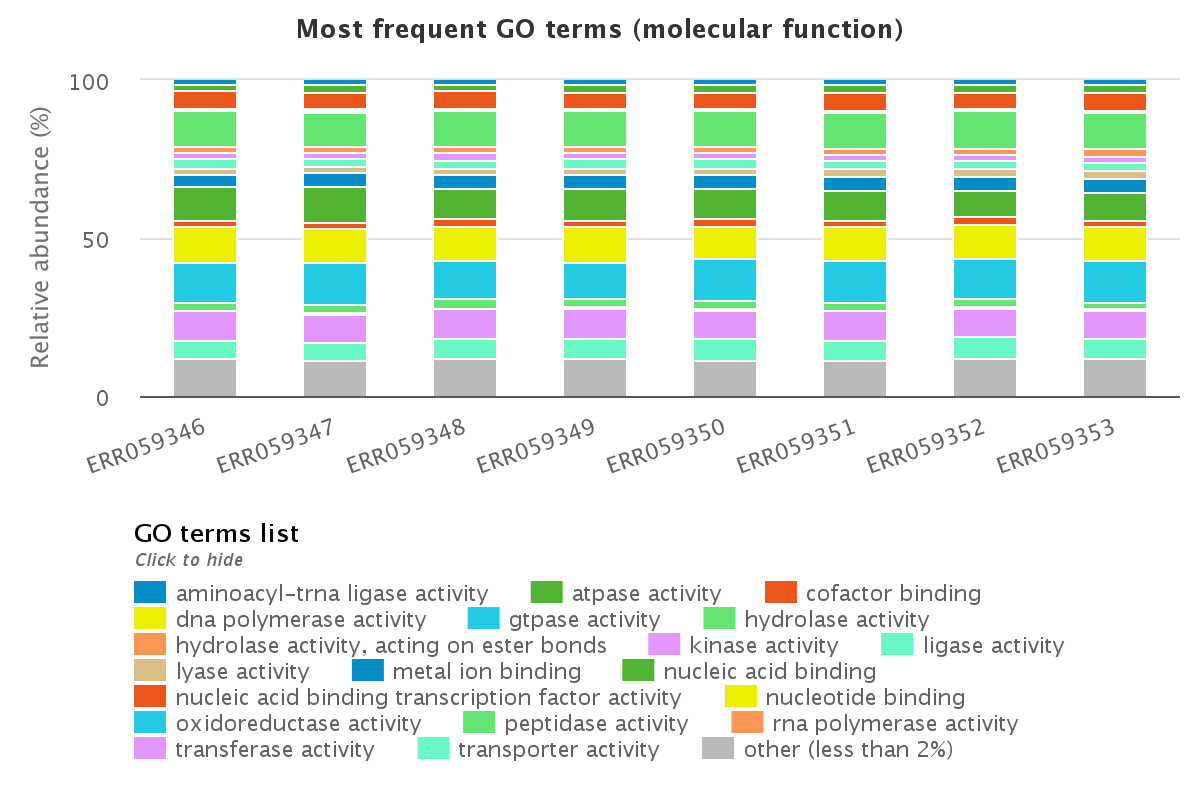


(B)


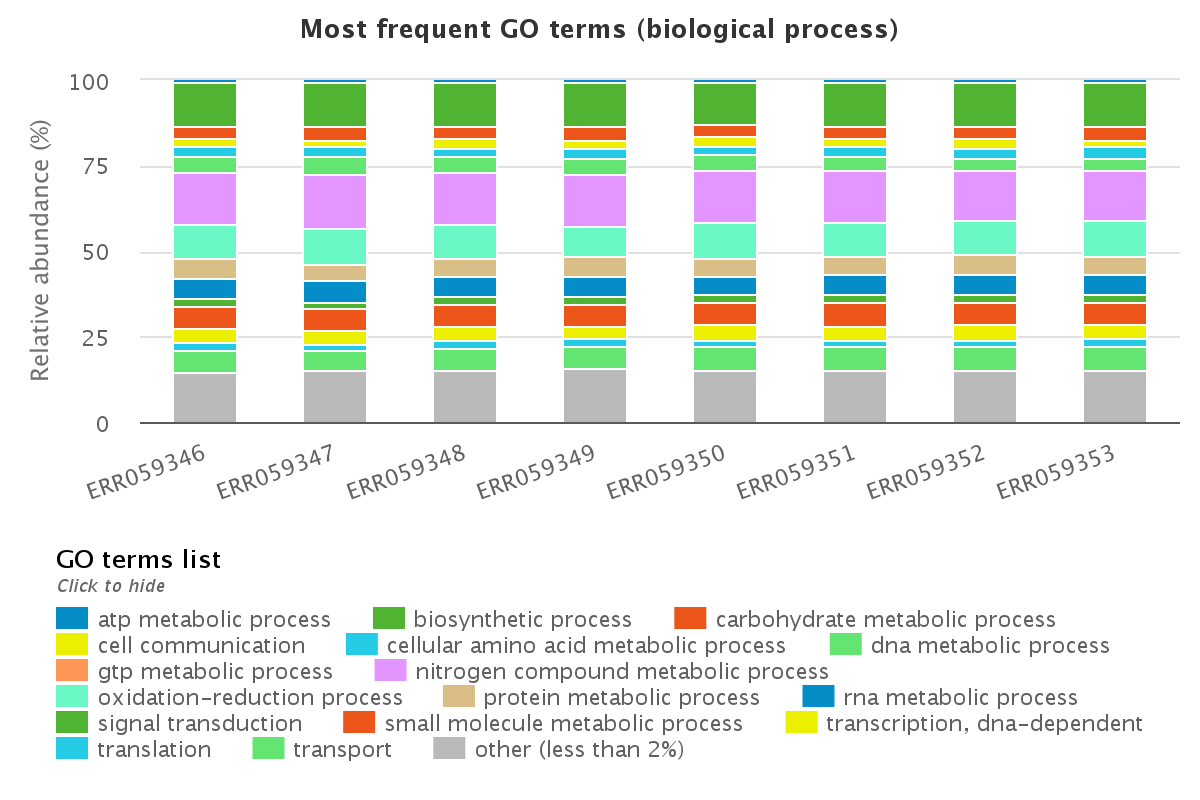


(C)


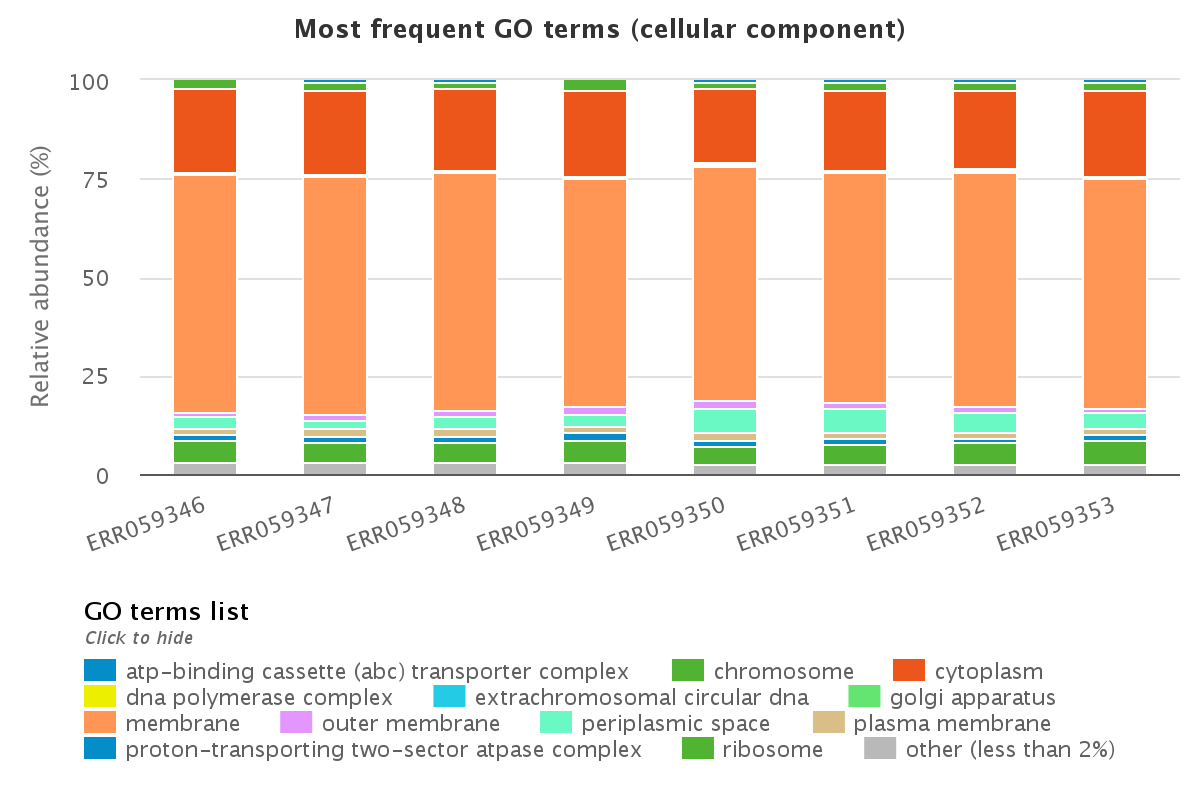


Figure S3

PhoX


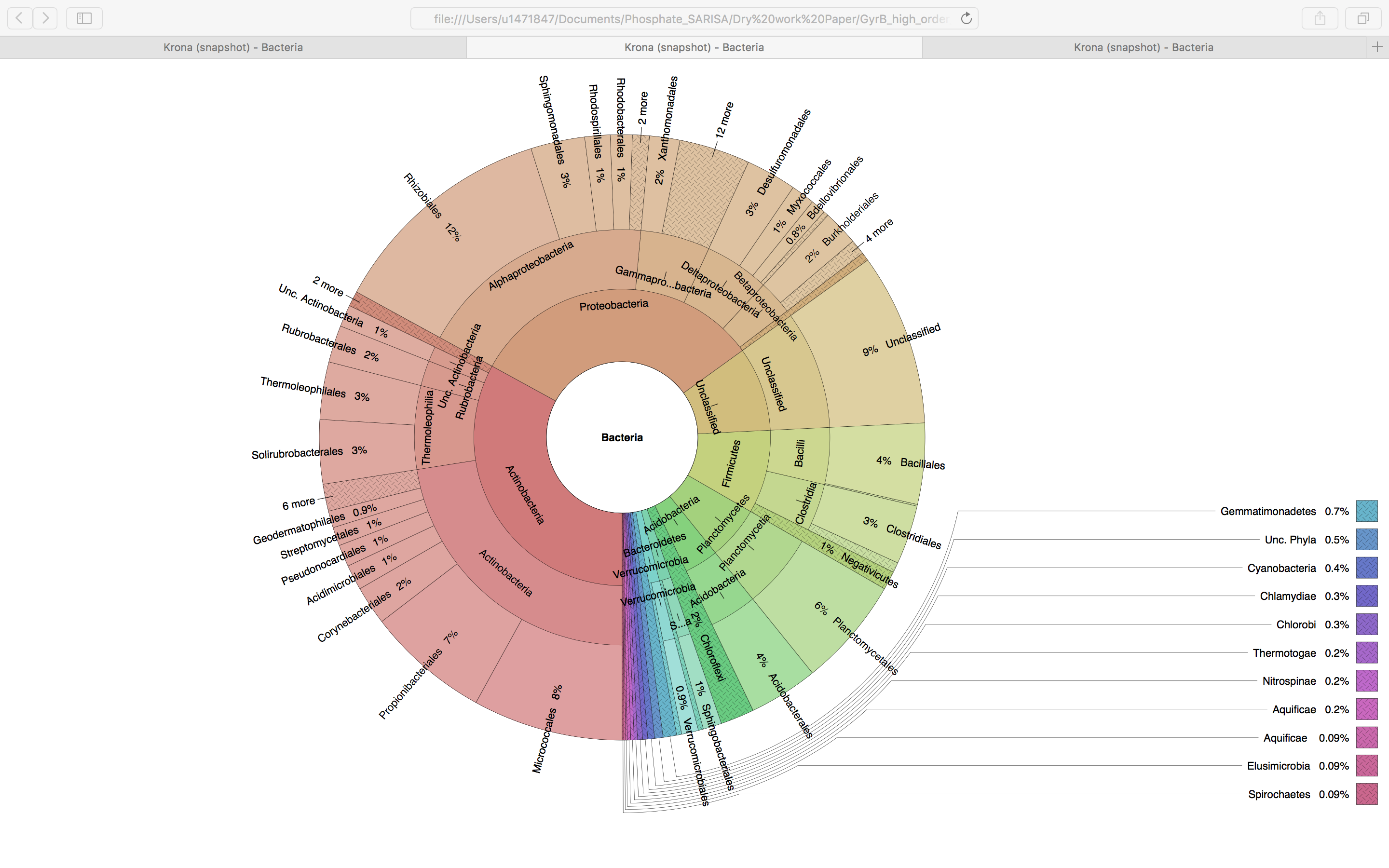
(A)


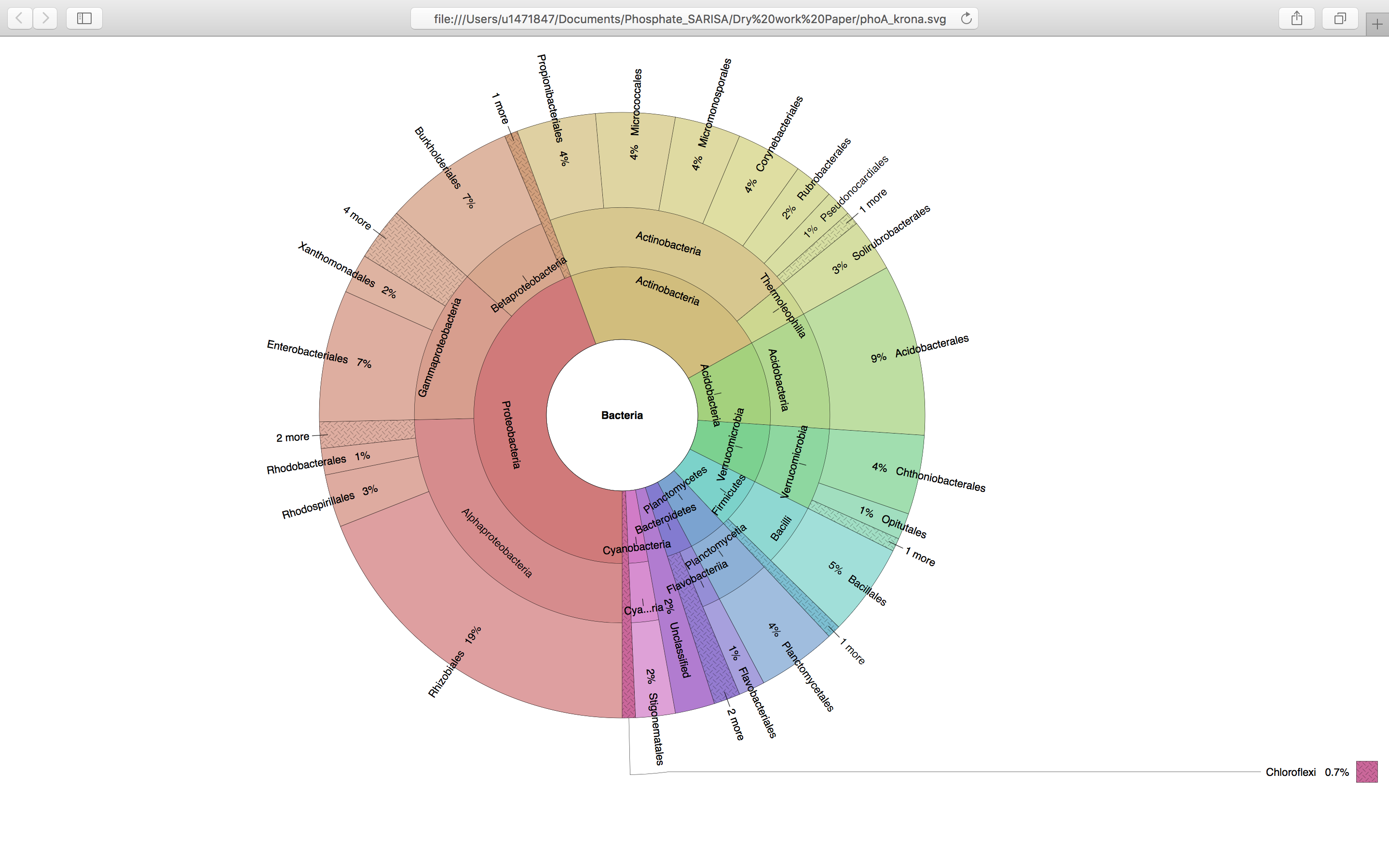
B)

PhoD

(C)

PhoA


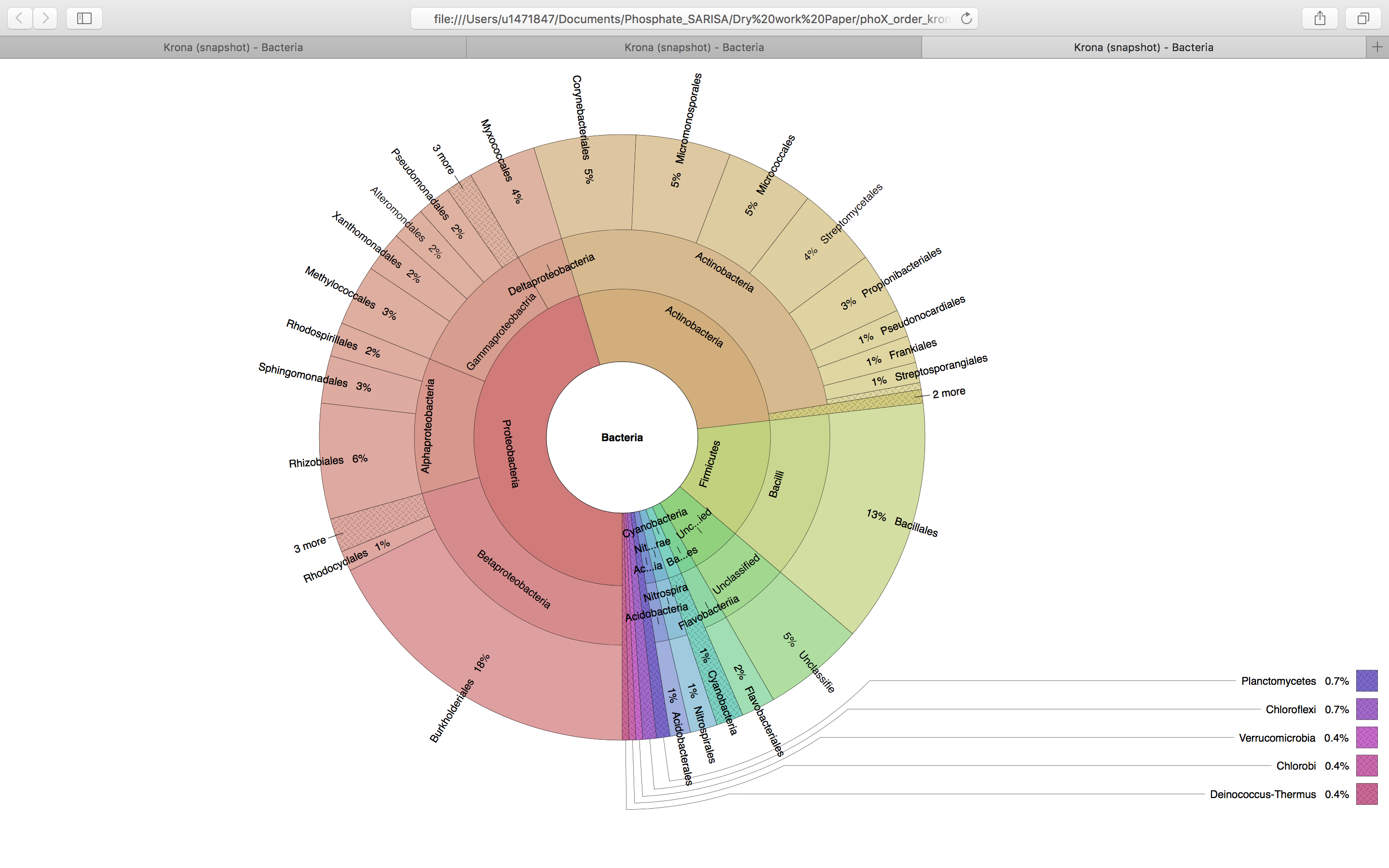


**Figure S4**

**
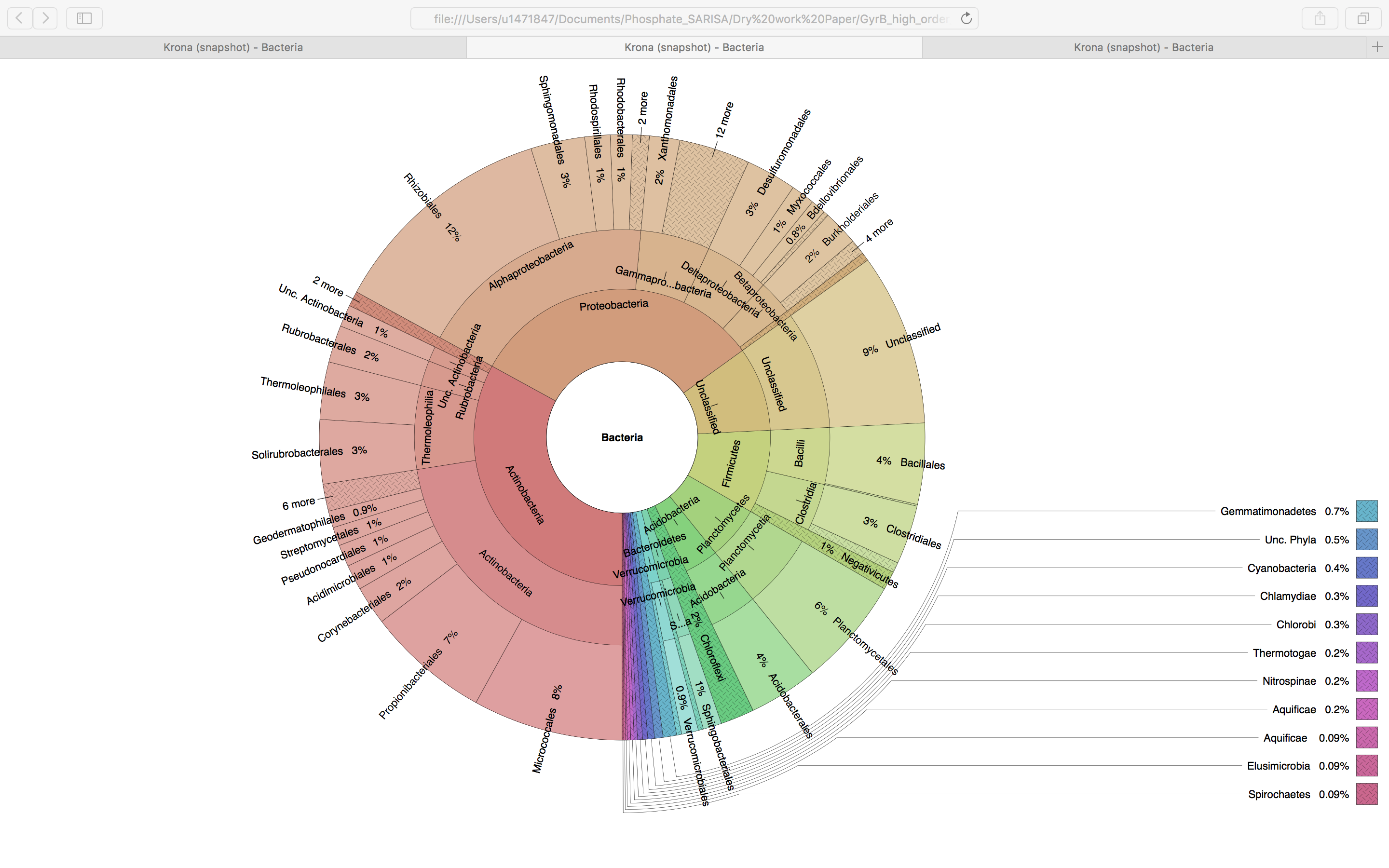
**

GyrB – high pH

**(A)**

GyrB – low pH

**
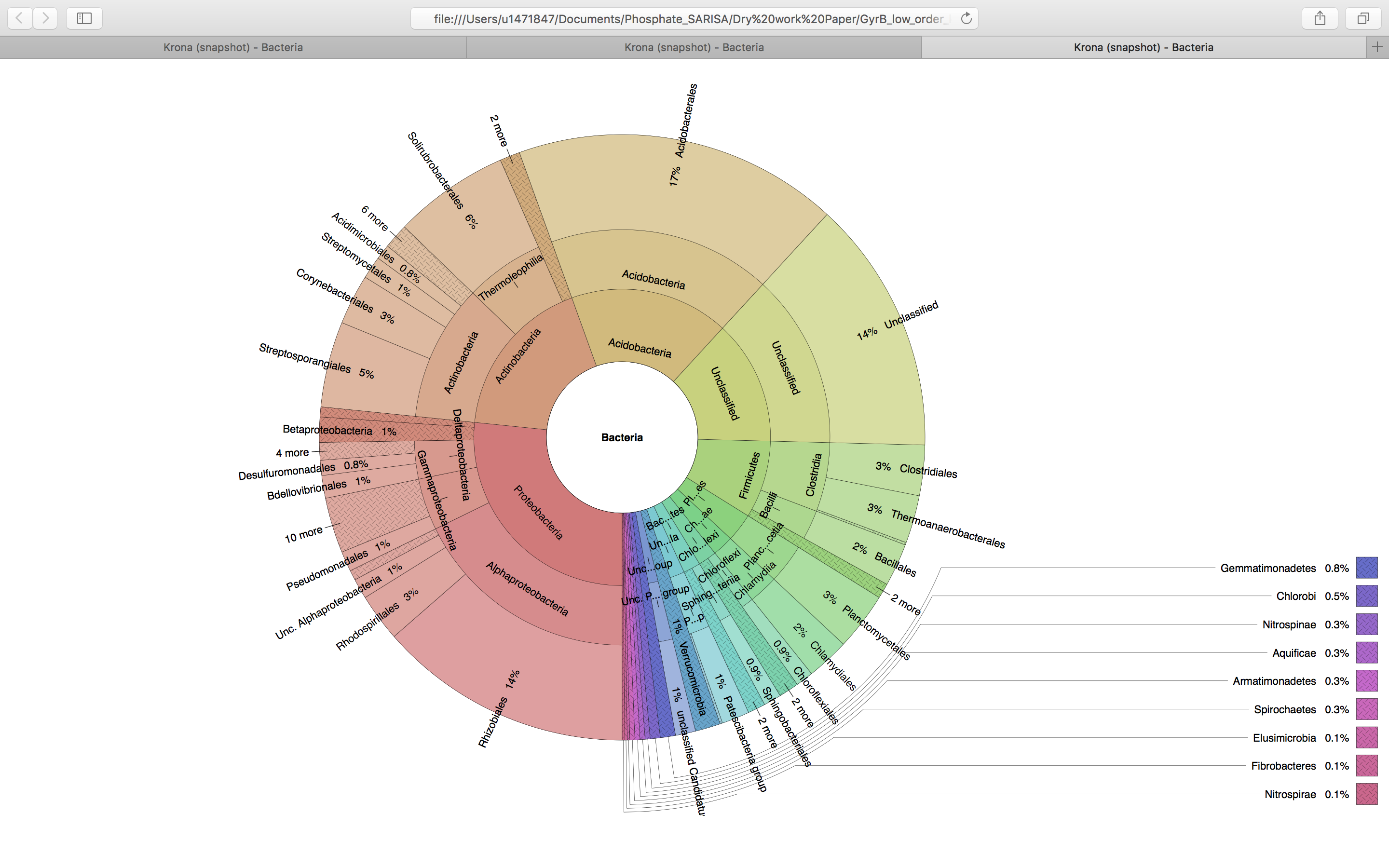
(B)**

**References**

Eddy SR (1998). Profile hidden Markov models. *Bioinformatics* **14:** 755-763.

Ondov BD, Bergman NH, Phillippy AM (2011). Interactive metagenomic visualization in a Web browser. *BMC Bioinformatics* **12:** 1-10.
